# Characterizing alternative splicing effects on protein interaction networks with LINDA

**DOI:** 10.1093/bioinformatics/btad224

**Published:** 2023-06-30

**Authors:** Enio Gjerga, Isabel S Naarmann-de Vries, Christoph Dieterich

**Affiliations:** Section of Bioinformatics and Systems Cardiology, Klaus Tschira Institute for Integrative Computational Cardiology, University Hospital Heidelberg, Heidelberg 69120, Germany; Department of Internal Medicine III (Cardiology, Angiology, and Pneumology), University Hospital Heidelberg, Heidelberg 69120, Germany; German Centre for Cardiovascular Research (DZHK), Partner Site Heidelberg/Mannheim, Heidelberg 69120, Germany; Section of Bioinformatics and Systems Cardiology, Klaus Tschira Institute for Integrative Computational Cardiology, University Hospital Heidelberg, Heidelberg 69120, Germany; Department of Internal Medicine III (Cardiology, Angiology, and Pneumology), University Hospital Heidelberg, Heidelberg 69120, Germany; German Centre for Cardiovascular Research (DZHK), Partner Site Heidelberg/Mannheim, Heidelberg 69120, Germany; Section of Bioinformatics and Systems Cardiology, Klaus Tschira Institute for Integrative Computational Cardiology, University Hospital Heidelberg, Heidelberg 69120, Germany; Department of Internal Medicine III (Cardiology, Angiology, and Pneumology), University Hospital Heidelberg, Heidelberg 69120, Germany; German Centre for Cardiovascular Research (DZHK), Partner Site Heidelberg/Mannheim, Heidelberg 69120, Germany

## Abstract

**Motivation:**

Alternative RNA splicing plays a crucial role in defining protein function. However, despite its relevance, there is a lack of tools that characterize effects of splicing on protein interaction networks in a mechanistic manner (i.e. presence or absence of protein–protein interactions due to RNA splicing). To fill this gap, we present Linear Integer programming for Network reconstruction using transcriptomics and Differential splicing data Analysis (LINDA) as a method that integrates resources of protein–protein and domain–domain interactions, transcription factor targets, and differential splicing/transcript analysis to infer splicing-dependent effects on cellular pathways and regulatory networks.

**Results:**

We have applied LINDA to a panel of 54 shRNA depletion experiments in HepG2 and K562 cells from the ENCORE initiative. Through computational benchmarking, we could show that the integration of splicing effects with LINDA can identify pathway mechanisms contributing to known bioprocesses better than other state of the art methods, which do not account for splicing. Additionally, we have experimentally validated some of the predicted splicing effects that the depletion of HNRNPK in K562 cells has on signalling.

## 1 Introduction

Alternative splicing (AS) contributes to the structural and functional diversity of proteins and might affect up to 95% of human gene loci (Wang et al. 2008). AS generates alternative mRNAs from precursor RNA sequences (pre-mRNA) by joining different combinations of exons. Different modes of AS exist and Exon Skipping (ES) is the most common among them (Wang et al. 2017). In ES, exons within the coding sequences may be skipped yielding alternative protein sequences upon mRNA translation. As such, AS processes may have various consequences on protein interactions through the removal or addition of protein interaction domains, thus making ES an important factor contributing to the modulation of signalling pathway activities as well as playing a significant role in gene expression. Nevertheless, despite their relevance, AS/ES effects are typically neglected when it comes to the functional and mechanistic analysis of signalling pathways.

Recent efforts (Louadi et al. 2021b) have led to the development of DIGGER as an online platform that provides an isoform-, domain-, and exon-specific view of protein interactions in humans. This makes DIGGER a suitable resource to support the development of computational methods that are used to characterize AS/ES effects on protein interaction networks. Following this line, NEASE (Louadi et al. 2021a) was further developed as a method used for the systematic analysis of the functional effects of AS. NEASE runs a hypergeometric test over interactions affected by protein splicing for gene set overrepresentation analysis (ORA). The value of NEASE was shown in several applications where it helped in identifying pathways contributing to tissue identity (muscle and neural tissues) as well as in highlighting aberrant splicing in diseases.

Despite their proven utility, enrichment-based methods like NEASE provide only a limited view on the effects of splicing upstream or downstream to the affected proteins. A more complete mechanistic understanding of splicing consequences on pathways and protein interactions would be of value to computationally predict effects in gene expression, cellular development as well as drug response. Two splice-agnostic ways to reconstruct protein interaction networks from transcriptomics are CausalR (Bradley and Barrett 2017) and CARNIVAL methods (Melas et al. 2015; Liu et al. 2019). CausalR was the first open-source causal network analysis platform used to predict the root cause of observed gene expression patterns by using causal reasoning principles. This was achieved through the implementation of an algorithm that scans for nodes with sign-consistent shortest paths to the observations. CARNIVAL, on the other hand, is an approach through which we can infer a subset of functional regulatory mechanisms upstream of regulated transcription factors (TFs) from prior knowledge of possible protein interactions. CARNIVAL typically relies on OmniPath (Türei et al. 2021) as a source of prior knowledge. However, the interactions reported in CARNIVAL and CausalR are typically verified through experiments involving only pairs of major protein isoforms. Additionally, none of the aforementioned resources maps individual exons to protein domains, which is essential for the consequent analysis of RNA splicing. In this way, the AS effects are largely neglected.

To address the above-mentioned limitations, we have developed Linear Integer programming for Network reconstruction using transcriptomics and Differential splicing data Analysis (LINDA) as the first tool for mechanistic characterization of splicing effects on protein interaction networks. Similar to CARNIVAL, LINDA aims to infer functional regulatory mechanisms upstream of functional TFs (Garcia-Alonso et al. 2021). However, to account for splicing effects, LINDA additionally integrates information about differentially spliced events/transcript abundance as well as available knowledge of domain-centric protein–protein interactions (PPIs). In summary, LINDA provides potentially useful insights about how splicing specifically affects cellular signalling in a specific context. We demonstrate the usefulness of LINDA in highlighting splicing effects in protein interaction networks as well as in identifying pathways associated with observed phenotype upon the silencing of target genes in K562 immortalized myelogenous leukaemia cells in an independent study (Replogle et al. 2022). Additionally, in one case example, we show the usefulness of LINDA in identifying the underlying regulatory mechanisms explaining how the depletion of the Heterogeneous Nuclear Ribonucleoprotein K (HNRNPK) protein affects splicing and consequently PPIs, some of which were experimentally validated.

## 2 Materials and Methods

### 2.1. Approach

**Figure 1.**
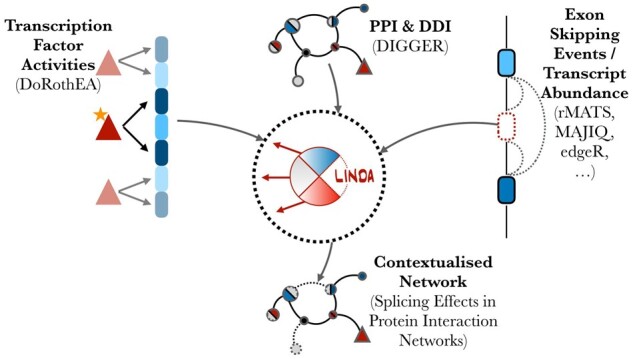
LINDA workflow. Prior knowledge of PPI and DDI networks is combined with TF activity scores and differential ES events/transcript abundances in order to infer functional interaction mechanisms. LINDA integrates all the three above-mentioned methodologies in order to infer regulatory interactions from functional domains (solid arrows) and abrogate interactions from skipped domains (dashed lines).

LINDA integrates information from prior biological knowledge on protein interactions and TF activities with RNA-seq data to infer contextualized cellular networks. Figure 1 depicts the LINDA workflow. The key components of the method are as follows: (i) From (differential/sample-wise) gene expression data, we estimate TF activities by using the DoRothEA (Garcia-Alonso et al. 2021) resource and the VIPER enrichment method (Alvarez et al. 2016). This step defines a set of significantly regulated TFs. LINDA aims to identify potential upstream mechanisms leading to the regulation of these TFs; (ii) We identify AS/ES events by analysing RNA-seq data through established computational methods (Mehmood et al. 2020) and map the respective transcript isoforms to their corresponding protein records and Pfam domains; and (iii) We build up a hypothesis space of possible interactions using DIGGER, which provides knowledge about PPI and domain-domain interaction (DDI) in a structured database.

LINDA will infer functional regulatory mechanisms between domains/proteins connecting an upstream regulator of signalling to downstream TFs. By default, LINDA adds to DIGGER an auxiliary ‘Perturbation’ which is connected with those genes in the resource which do not have any upstream regulator. In a typical analysis workflow we start by estimating TF activities from (differential) gene expression data using the DoRothEA package (Garcia-Alonso et al. 2021). LINDA then applies a Causal Reasoning approach to infer a subset of protein–protein and domain–domain regulatory mechanisms which are responsible for the regulation of downstream TFs. LINDA uses the DIGGER resource of PPI and DDI. This means that the functional interactions inferred by LINDA, will represent a subset of the interactions present in DIGGER.

Our novel contribution is to integrate information about splicing events into this approach. For this, we can either rely on (i) methods used for the identification of differentially skipped exons; or (ii) methods to pinpoint differential transcript abundance. There are numerous methods to identify differential splicing events like rMATS (Shen et al. 2014) and MAJIQ (Vaquero-Garcia et al. 2016). For exon-skipping events, we require size-effect and significance estimates which hold information about the difference in the inclusion/exclusion levels of a specific exon in the final mRNA transcript between two samples or conditions we want to compare. Typically, effect sizes such as dPSI parameter inform us of the direction of change as well (i.e. exclusion versus inclusion in a comparison of two conditions). A negative dPSI value indicates an exon exclusion event while a positive dPSI value indicates an exon inclusion event relative to a control condition. DIGGER provides mapping tables between exons, corresponding protein isoforms and, Pfam domains. Thus, we can then identify skipped protein domains based on the evidence from differential ES data. A protein domain will be considered as skipped if the set of exons to which this protein domain maps to are also considered to be skipped (Supplementary Fig. S1). Skipped exons in this case are considered those whose *P*-value falls below a user-defined threshold. DIGGER also provides mapping tables between transcript identifiers and corresponding protein Pfam domains, which enables differential transcript abundance analysis with the aim to identify skipped protein domains. In this case, however, the size effect represents the log-fold-change (logFC) value in the transcript abundance difference between the two conditions that we are comparing. Similar to dPSI, the sign of the logFC value will tell for the direction of change and the logFC values are associated with a *P*-value obtained from applying various significance tests. We can rely on methods such as edgeR (Robinson et al. 2010) to estimate differentially expressed transcripts between two conditions from RNA-Seq transcript abundance data. In the case, when we have multiple exons/transcripts mapping into a single specific domain of a protein, the *P*-value, which indicates how confident we are about whether this domain is to be considered as skipped is determined by performing a Fisher’s aggregation procedure (Lancaster 1961) over all the *P*-values of the corresponding exons and transcripts. The size effect scores assigned to the protein domains are then simply calculated as the average among the size effect scores (dPSI/logFC) of the matching exons/transcripts.

LINDA then uses a deterministic global optimization approach which aims to identify the subset of functional elements (proteins, domains, or interactions between them) from the knowledge available in DIGGER. Each of these elements has been assigned a discrete logic variable (0 or 1), which represents the state that these elements can take. Such a discrete representation of the state variables allows the formulation of the network-building process of LINDA as an Integer Linear Problem (ILP), which is defined by a linear objective function as well as a set of linear constraints. In LINDA, the objective function aims to prioritize networks, which include TFs that are estimated to be most significantly active, while penalizing the inclusion of those TFs that are not considered to be regulated. A second term of the objective function penalizes the size of the solution (regularization) to reduce the number of domain/protein elements to be included in the solution. The set of linear constraints on the other hand defines the functional relationships among the discrete variables. We use constraints to define the rules by which domain and protein components are allowed to interact with each other. The constraints also define the rules of possible functional interactions based on evidence from splicing events or the identified skipped domains. Such rules can e.g. enforce that a specific DDI would be considered to be non-functional if at least one of the domains in the interacting pairs is skipped. On the other hand, an interaction between two proteins would only be possible if at least one of its constituent DDIs is inferred to be functional.

There are two modes in which the users can run the LINDA-splice-aware analysis: the hard-constrained and the soft-constrained modes. In the first approach, skipped exons are considered those whose *P*-value falls below a threshold specified by the user. In this way, hard constraints are set to the ILP formulation of LINDA in order to ensure that interactions involving skipped domains (mapped to the skipped exons/transcripts) are considered as non-functional. On the soft-constrained approach, on the other hand, no hard constraints are set to the ILP formulation but instead, exon/domain skipping significance scores are added as another penalization term in the objective function. In this case, the level of the penalization will depend on the level of the significance of the ES event (proportional to -log(*P*-value)). Mathematical details about the ILP formulation for LINDA for both modes of analysis can be found in the Supplementary materials (Supplementary Text S1).

### 2.2 Tools and Resources

#### 2.2.1 LINDA

The network reconstruction approach relies on an ILP-based optimization technique. For more details, please refer to Supplementary Text S1.

#### 2.2.2 DoRothEA

DoRothEA is a resource of TF to gene target relations with various levels of confidence (A, B, C, D, and E) from which we can estimate the highly regulated TFs based on the expression of their target genes. The TF regulation levels are inferred via VIPER (Alvarez et al. 2016). In our analysis, we also performed statistical testing to estimate the significance of the enrichment scores through a permutation analysis. This was achieved by randomizing the bi-partite TF-to-Gene graph from DoRothEA 1000 times (Milo et al. 2003; Iorio et al. 2016). The significant *P*-value is then estimated by looking at how extreme the originally estimated TF activity is to the null distribution of scores we generate. Significantly regulated TFs are considered to be those that pass the significance threshold *P*-value ≤ 0.05 from our permutation analysis.

#### 2.2.3 DIGGER

The DDIs and PPIs were combined with the tables mapping the transcripts and exons to the corresponding Pfam domains and genes that DIGGER provides. In this way, we have obtained a single comprehensive table that provides information about the interacting domain (with Pfam identifiers) and protein (with conventional gene names) components as well as about the Transcript and Exon Ensembl identifiers into which these domains are mapping. In total, we get a table of 88 732 unique interactions for humans which can also be automatically loaded from the LINDA package.

#### 2.2.4 ENCORE

ENCORE is a sub-project of ENCODE (https://www.encodeproject.org/encore-matrix) whose goal consists of identifying protein–RNA interactions by creating a map of RNA binding proteins (RBPs) encoded in the human genome and identifying the RNA elements that the RBPs bind to. It consists of a large resource of ˃20 000 experiments, including shRNA KD of over 200 genes and RNA-seq experiments.

#### 2.2.5 CARNIVAL

CARNIVAL (Liu et al. 2019) is a method used to contextualize signed and directed protein interaction networks from TF activities based on causal reasoning principles.

#### 2.2.6 CausalR

CausalR represents another causal reasoning network contextualization tool. For our benchmarking studies, path lengths from one to five edges were scanned and the potentially dysregulated nodes were identified as those which constantly score among the top 100 based on the number of explained observations.

#### 2.2.7 HepG2 cell culture and siRNA-mediated reduction of HNRNPK

HepG2 cells (German Collection of Microorganisms and Cell Cultures GmbH, ACC 180) were cultured in DMEM + 10% FCS + 1× penicillin/streptomycin at 37°C and 5% CO_2_. Cells were transfected with RNAiMAX and siRNAs directed against HNRNPK (Naarmann et al. 2008) or a non-targeting control siRNA (Thermo Fisher Scientific) according to the manufacturer's protocol and harvested 48-h post-transfection. Cytoplasmic and nuclear extracts were prepared according to the method published by Dignam et al. (1983). Extracts were stored at −80°C until further usage. Protein concentration was determined by Bradford measurement (RotiNanoquant, Carl Roth).

#### 2.2.8 RNA isolation and RT-qPCR

RNA was isolated with Trizol (Thermo Fisher Scientific) according to the manufacturer’s protocol and reverse transcribed with random primers using the Maxima First strand cDNA synthesis kit (Thermo Fisher Scientific). The HNRNPK mRNA level was determined by RT-qPCR as described (Naarmann-de Vries et al. 2019).

#### 2.2.9 Polyacrylamide gel electrophoresis and Western blotting

Proteins were separated on 4–12% Criterion XT Bis–Tris gels (Bio-Rad) using XT-MES (Bio-Rad) as running buffer. They were blotted onto a PVDF membrane (Millipore) in 1× Tris-glycine buffer (Bio-Rad) + 10% ethanol. Antibodies were incubated according to Supplementary Table S3. Western blot signals were detected after incubation with horseradish peroxidase-coupled secondary antibodies using SuperSignal West Pico and Atto chemiluminescent substrates (Thermo Fisher Scientific) and a ChemiDoc system (Bio-Rad). Images were quantified with Image Lab (Bio-Rad) and further processed with ImageJ.

#### 2.2.10 Immunoprecipitation of MAPK3

Erk1 (MAPK3) was immunoprecipitated as described previously (Naarmann et al. 2008) from nuclear extracts employing a specific Erk1 (MAPK3) antibody (Abcam, ab32537), and Dynabeads Protein A beads (Thermo Fisher Scientific).

## 3 Results

The ENCORE project (https://www.encodeproject.org/encore-matrix) provides experimental data targeting RNA-binding proteins by Small hairpin (shRNA)-mediated gene silencing over the Hepatocellular Carcinoma (HepG2) and Chronic Myelogenous Leukaemia (K562) cell-line. To this end, we have obtained the read counts for gene and transcript abundance values for two biological replicates in two types of experimental data or conditions: (i) RNA-Seq on HepG2 and K562 cells transfected with shRNAs directed against target genes (from hereon referred to as knockdown [KD]); and (ii) Control shRNA against no target in HepG2/K562 cells followed by RNA-seq (here on referred to as Ctrl). Differential gene and transcript abundances were then estimated for each individual gene and transcript by comparing KD-versus-Ctrl conditions for each perturbation target and each cell type separately by using edgeR (Robinson et al. 2010).

Differential gene expression (DGE) data from the KD-versus-Ctrl comparisons were used to estimate differential TF activities with DoRothEA. Significantly regulated TFs were considered those that pass the significance threshold *P*-value 0.05 from the implemented permutation analysis (see Methods section). The network-building process from LINDA was then used to infer the upstream processes leading to the regulation of those TFs which are considered to be significantly regulated, while penalizing the inclusion of those TFs which are not significant. Besides DGE’s we additionally computed differential transcript expression for the KD-versus-Ctrl comparisons. Since DIGGER provides a list of transcript identifiers mapped into the domains of each protein, this allows us to make use of the estimated differential transcript abundances in order to identify which of the domains is to be considered as skipped for each protein available in DIGGER.

### 3.1 Splicing effects as a consequence of HNRNPK-KD contribute to interaction rewiring in HepG2 cells

We have applied hard-constrained LINDA in order to evaluate the protein interaction networks upon the KD of 47 genes in the HepG2 cell line based on the data provided by ENCORE. In all the cases, the networks were analysed by taking into account the splice effects made evident from the differential transcript analysis (splice-aware analysis) as well as without taking into account such effects (splice-unaware). The two types of networks were then combined with each other in order to make a comparison between them as well as to highlight better the splicing effects in the protein interaction networks. As a demonstrative example, we focus on HNRNPK shRNA KD experiments. This well-studied RNA-binding protein has been demonstrated to regulate the splicing of a specific set of target genes (Revil et al. 2009; Thompson et al. 2018; Escobar-Hoyos et al. 2020). From HNRNPK shRNA KD experiments we try to recapitulate the KD effects in the rewiring of functional PPIs. In this case application, LINDA enumerated 100 diverse network solutions in the splice-unaware analysis and 96 solutions in the splice-aware (hard-constrained mode) analysis after letting CPLEX run for 2 h and using 20 threads. The diverse solutions were integrated as a single network (separately in splice-aware and splice-unaware networks) where each DDI was assigned a weight value between 0 and 1 indicating how often an interaction appears across the diverse networks retrieved. Such integration allows making a better comparison between the splice-aware and splice-unaware networks as well as highlighting better the splicing effects in the protein interaction networks. The resulting combined network contains 116 DDI’s between 81 domains and 57 unique proteins. Of the 81 domains reported in the integrated network, 27 were identified as skipped after splice-aware analysis.

**Figure 2.**
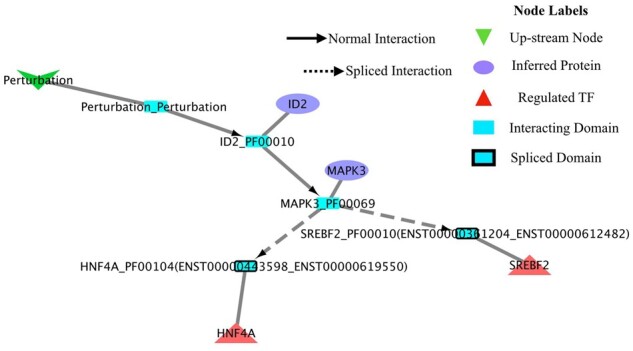
Set of interactions functional DDI’s inferred exclusively in the LINDA splice-unaware analysis and not in the splice-aware approach upon HNRNPK depletion. The small quadratic nodes depict the interacting domains labelled with the corresponding Gene and Pfam domain ID. The small blue nodes highlighted with the black borders represent the spliced domains while with dashed lines are depicted the interactions in which at least one of the domain partners is spliced. In brackets of the spliced domain labels are shown transcript ID’s mapped to their corresponding domains.

We noticed an interesting difference in our comparative analysis of the splice-aware and splice-unaware networks for the HNRNPK perturbation case. Figure 2, highlights the relevant connections from the ‘Perturbation’ node to the downstream SREBF2 and HNF4A TFs. All interactions were inferred in the splice-unaware solution but not in the splice-aware network. More specifically, the two TFs appear to be regulated by MAPK3 (Erk1) kinase, which in turn is affected by its upstream regulator ID2 as a result of the interactions between the Helix-loop-helix DNA-binding domain of ID (PF0010) and the Protein kinase domain (PF00069) of MAPK3. In turn, MAPK3 appears to regulate the two downstream TFs through it’s the interaction of PF00069 MAPK3 domain with the Ligand-binding domain of nuclear hormone receptor (PF00104) domain of HNF4A and PF00010 domain of SREBF2 protein. Both of these DDI’s (PF00069 → PF00104 of MAPK3 → HNF4A and PF00069 → PF00010 of MAPK3 → SREBF2) are interactions that appear to be disrupted when accounting for splicing effects as domains PF00104 of HNF4A and PF00010 of SREBF2 are revealed to be skipped as a result of splicing.

**Figure 3.**
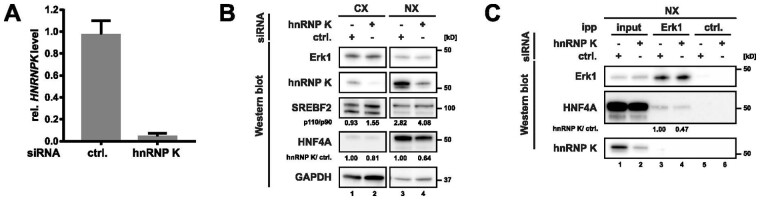
HepG2 cells were transfected with siRNAs directed against hnRNP K or a non-targeting control (ctrl.) siRNA. A) RT-qPCR analysis of HNRNPK mRNA levels in ctrl. and HNRNPK depleted HepG2 cells normalized to ACTB mRNA. B) Western blot analysis of MAPK3, HNRNPK, SREBF2, HNF4A, and GAPDH mRNA levels in cytoplasmic (CX) and nuclear extracts (NX). The ratio of the two main SREBF2 isoforms in every sample as well as the level of HNF4A in HNRNPK depleted cells versus ctrl. cells were quantified. Shown is the mean value from two independent blots and three exposure times each. C) MAPK3 was immunoprecipitated from nuclear extracts as indicated (lanes 3–4). The successful precipitation of MAPK3, as well as a co-precipitation of HNF4A and HNRNPK, was analysed in Western blots. The amount of co-precipitated HNF4A in HNRNPK versus ctrl. depleted cells was quantified.

In order to validate these predictions, we depleted HNRNPK from HepG2 employing siRNA transfection (Fig. 3A and B). While depletion of HNRNPK had no direct effect on the expression of MAPK3 (Fig. 3B), we interestingly observed an isoform change for SREBF2 (Fig. 3B). Especially in the cytoplasmic fraction (CX), a shift from the smaller to the larger SREBF2 isoform was detected (Fig. 3B). Furthermore, the main isoform of HNF4A is reduced in HNRNPK-depleted cells (Fig. 3B). Additional isoforms of HNF4A are near the detection limit and could not be accurately quantified. Next, we immunoprecipitated MAPK3 from nuclear HepG2 extracts (NX), as HNF4A is mainly localized in this compartment. Importantly, we were able to detect the predicted interaction of MAPK3 with HNF4A, and found a reduced interaction upon HNRNPK depletion (Fig. 3C). The validation of a differential SREBF2 interaction was unfortunately not possible due to the crossreactivity of the antibody.

### 3.2 Computational benchmarking on the K562 ENCORE dataset

In order to evaluate the global relevance of introducing information about splicing effects in protein interaction networks, we have used the pool of LINDA networks generated for the K562 cells from ENCORE and aimed to do a comparison with the other state-of-the-art and similar methods, namely CARNIVAL and CausalR. For such a benchmark, we have additionally relied on independent large-scale Perturb-seq experiments using CRISPRi study in which all expressed genes in K562 chronic myeloid leukaemia cells were targeted (*n* = 9876 targets repressed) (Replogle et al. 2022). In this study, unbiased clustering of similar perturbations within the dataset was performed where 64 discrete clusters were identified and their functions were annotated by using CORUM (Ruepp et al. 2008), STRING (Szklarczyk et al. 2019), and manual searches. Such clusters and embeddings showed a clear organization by biological function spanning an array of processes and which can be assigned to a corresponding gene ontology (GO) term (Ashburner et al. 2000). Before performing computational benchmarking, as a first step, we have identified overlaps between the ENCORE experiments and the CRISPRi targets of the Perturb-Seq study (Replogle et al. 2022) where a total number of 24 genes were identified as targets for both of the studies. Then we identified, which ontological is associated with each repressed genes in the Perturb-Seq analyses—From the 24 targets, 16 cases were unambiguously associated with a phenotype, namely: HNRNPA2B1, HNRNPC, HNRNPU, MAGOH, NCBP2, PABPN1, PAPOLA, PCBP1, POLR2G, PPIL4, PRPF6, PRPF8, PUF60, SF1, SRSF1, and SMNDC1. And finally, for each of the phenotypes associated with the perturbation of these 16 genes, we identify a corresponding pathway set from the GO collection of pathways by using the AmiGO web-service (Carbon et al. 2009). A table of perturbation targets in K562 clustered to each of their specific phenotypes and their corresponding GO pathway sets has been made available in Supplementary Table S2.

**Figure 4.**
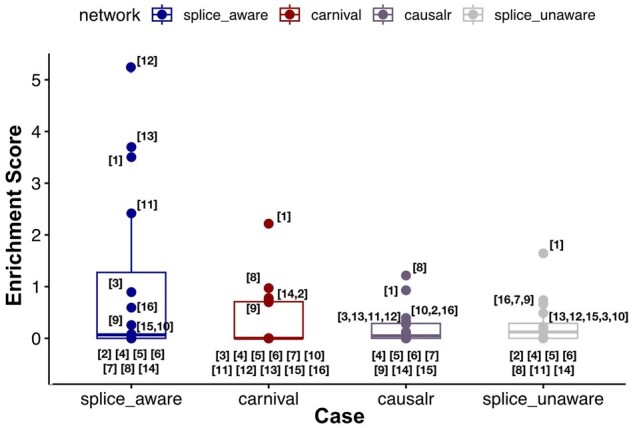
Comparing the distribution of enrichment scores: LINDA splice-aware versus CARNIVAL versus CausalR versus LINDA splice-unaware. Here are depicted and compared the pathway enrichment score values (as -log10(*P*-values)) of biological functions/phenotypes for LINDA (splice-aware and splice-unaware) and CARNIVAL. The splice-aware LINDA analyses were performed in the soft-constrained mode: 1. HNRNPA2B1—Histone acetylation; 2. HNRNPC—NuA4 histone acetyltransferase complex; 3. HNRNPU—Spliceosome; 4. MAGOH—Non-sense Mediated Decay; 5. NCBP2—mRNA Capping; 6. PABPN1—Exosome and mRNA turnover; 7. PAPOLA—Mediator Complex; 8. PCBP1—COP9 Signalosome; 9. POLR2G—Nucleotide Excision Repair; 10. PPIL4—Spliceosome; 11. PRPF6—Spliceosome; 12. PRPF8—Spliceosome; 13. PUF60—Spliceosome; 14. SF1—NuA4 histone acetyltransferase complex; 15. SMNDC1—Spliceosome; 16. SRSF1—Mediator Complex.

In this computational benchmarking approach, for each selected 16 gene perturbation experiments, we have decided to compare the LINDA splice-aware networks which we infer when using the soft-constraint mode with networks which we obtain when using LINDA-splice-unaware, CARNIVAL, and CausalR. Then for each network (CARNIVAL, CausalR, LINDA-splice-aware, and LINDA-splice-unaware) we perform an ORA of the nodes/proteins/genes inferred over the GO term sets with the fgsea R-package (Korotkevich et al. 2021). From the ORA analyses, we then estimate the enrichment scores (as -log10 of the *P*-values) for each GO term assigned to our genetic perturbation targets. The higher the enrichment score, the more enriched is the target GO term. Finally, we have compared the enrichment score distributions for the CARNIVAL, CausalR, LINDA splice-aware (soft-constrained), and unaware networks. Figure 4 shows a comparison about how well the splice-aware LINDA networks can identify signalling mechanisms which are expected to be enriched for each perturbation experiment compared to CARNIVAL.

As we can notice, compared to CARNIVAL and CausalR, the LINDA networks can significantly improve the gene-level enrichment analysis for those GO terms that match the phenotypic response of K562 cells on each selected perturbation. Indeed, a statistical testing analysis (paired Student’s *t*-test: (i) LINDA splice-aware versus CARNIVAL—*t* = 1.6485, *P*-value = 0.1187; (ii) LINDA splice-aware versus CausalR—*t* = 1.9313, *P*-value = 0.07136 and (iii) LINDA splice-aware versus LINDA splice-unaware—*t* = 1.9217, *P*-value = 0.07264 over the enrichment score distribution of target GO terms, showed that integration of information about splicing through LINDA improves the enrichment of target phenotypes compared to CARNIVAL, CausalR as well as the LINDA splice-unaware networks.

## 4 Discussions

In this article, we have described LINDA as a method that infers regulated protein interaction networks based on evidence from gene expression data and AS at the same time. As such, LINDA uncovers the potential effects of AS on cellular signalling networks. Similar to many other network analysis tools (Beisser et al. 2010; Gjerga et al. 2020, 2021; Llabrés et al. 2020), the network reconstruction process in LINDA is performed by the implementation of an Integer Linear Programming (ILP) formulation, which offers some obvious advantages such as the identification of global optimal solutions through the use of available efficient solvers. Additionally, the modelling process can be controlled by a wide range of optimization parameters and settings (see Supplementary Table S1). LINDA implements three types of solvers (COIN-Cbc, lpSolve, and the default CPLEX) and a comparison between such solvers has been provided in the Supplementary material (Supplementary Text S2).

LINDA extends a previous method called CARNIVAL (Liu et al. 2019). Similar to CARNIVAL, LINDA merges TF activities estimated from gene expression data with prior knowledge on the signalling network architecture to identify processes downstream of signalling which are driving changes in gene expression. However, one of the limitations of CARNIVAL is that it neglects AS due to the absence of this information in data analysis and corresponding resources of protein interactions. LINDA overcomes this limitation by relying on the DIGGER resource, which integrates PPI and DDI. In this way, LINDA is able to integrate the functional effects of alternative exon usage (as identified through various differential splicing tools or from differential transcript abundance analysis) over the structural composition of different protein isoforms and their ability to interact with other proteins. One disadvantage of LINDA, however, is that it is not able to identify the direction of the regulation of inferred proteins (up-/down-regulation) as CARNIVAL does. This is due to the fact that the DIGGER resource provides no information about the nature of the interaction between pairs of proteins (activatory/inhibitory interactions) or how splicing is able to affect signs of interactions. However, LINDA could be easily extended once RNA splicing resolved information on the direction of interaction becomes available. Currently, LINDA and CARNIVAL, as well as CausalR, should rather be seen as complementary to each other as both of these methods can be used to give answers to different biological questions.

Network reconstruction methods are able to reveal potential mechanisms of interactions between proteins. Through such a mechanistic approach, we are not only able to identify associations between biological molecules, but we can also reveal the underlying molecular mechanisms involved in such associations. This makes network inference tools especially useful to pinpoint potential therapeutic targets and to make predictions about the potential outcome of a treatment. However, signalling network reconstruction considering RNA splicing effects on the protein interactome is underrepresented in literature. We have tried to fill this gap with LINDA. This work is a start but delivers a promising hypothesis as exemplified by comparing splice-aware and splice-unaware networks.

## Supplementary Material

btad224_Supplementary_Data

## Data Availability

LINDA has been implemented as an R-package and it is available online in: https://dieterich-lab.github.io/LINDA/ along with the ENCORE data used, results as well as tutorials.
