# Supplementary material for: Characterizing alternative splicing effects on protein interaction networks with LINDA
Source: Bioinformatics. 2023 Jun 30;39(Suppl 1):i458–64. doi: 10.1093/bioinformatics/btad224 (PMC10311343; doi:10.1093/bioinformatics/btad224)
Supplement: btad224_Supplementary_Data [file btad224_supplementary_data.pdf]

Characterising Alternative Splicing Effects to Protein Interaction Networks with LINDA

Enio Gjerga[1,2,3], Isabel S. Naarmann-de Vries[1,2,3], Christoph Dieterich[1,2,3,#]

[1] Section of Bioinformatics and Systems Cardiology, Klaus Tschira Institute for Integrative Computational Cardiology, University Hospital Heidelberg, 69120 Heidelberg, Germany
[2] Department of Internal Medicine III (Cardiology, Angiology, and Pneumology), University Hospital Heidelberg, 69120 Heidelberg, Germany
[3] German Centre for Cardiovascular Research (DZHK) - Partner Site Heidelberg/Mannheim, 69120 Heidelberg, Germany

[#] Corresponding author: christoph.dieterich@uni-heidelberg.de

Supplementary Materials:

S-Fig-1 Effects of domain skipping in protein interactions 2
S-Text-1 ILP Formulation 3
S-Text-2 LINDA Solvers 10
S-Tab-1 Parameter Settings 11
S-Tab-2 GO Terms 14
S-Tab-3 Antibodies 15

S-Fig-1: Effects of Exon Splicing on Protein Domains.

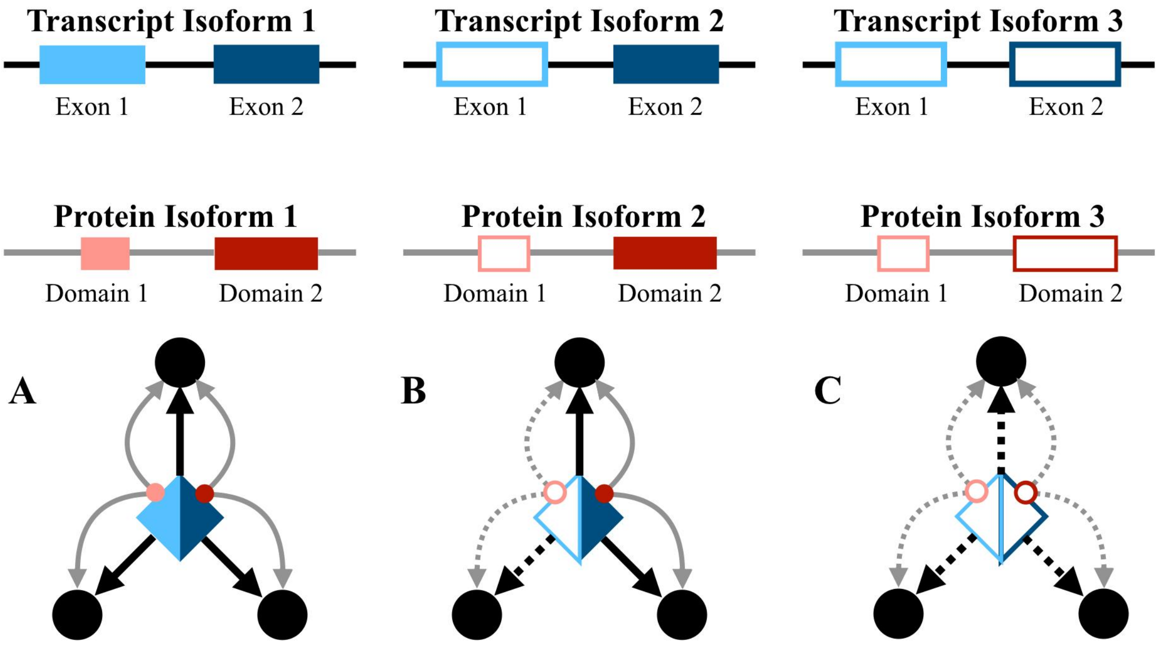

Thin grey arrows correspond to domain-domain interactions and thick black arrows correspond to interaction between proteins. Solid line shows that an interaction is not affected by domain skipping and dashed line depicts interactions affected by domain skipping. A) Exon1 (Domain1) and Exon2 (Domain2) are not skipped. All the three PPIs are plausible. B) Exon1 (Domain1) is skipped and Exon2 (Domain2) is not skipped. The protein interaction on the left becomes impossible while the other two interactions are still plausible. C) Exon1 (Domain1) and Exon2 (Domain2) are skipped. All the three PPIs will not happen.

S-Text-1: ILP formulation. LINDA takes information about Prior knowledge of protein-protein and domain-domain interaction Networks (PKN), combined with transcription factor activity scores and differential exon skipping events/transcript abundances as inputs in order to infer functional interaction mechanisms. Once the inputs are provided, the identification of the functional mechanisms from DIGGER the general PKN (linking the highly activated transcription factor targets to the upstream regulators) is performed via the implementation of an Integer Linear Programming (ILP) formulation. These functional mechanisms include the directed interactions and upstream signalling proteins that are most likely to be present in order to explain the observed gene expression patterns and splicing events.

ILP is a mathematical optimization problem in which the objective function and constraints are linear, while the variables are integers. The general statement of an ILP problem with n variables and m inequality equations can be defined as follows:

$$\text{Objective: } min(c^T x)$$

$$\text{Subject to: } Ax \leq b, \quad x \in Z^n$$

$$\text{Bounded by: } l \leq x \leq u,$$

From the above definition, with x we refer to the optimization variable and we want to maximize a linear function $c^T x$ ($c \in R^n$) on a region defined by the intersection of half-plains $S = \{x | Ax \leq b,\ l \leq x \leq u\}$. The x is an n vector of unknown integers ($x \in Z^n$) which we want to identify or estimate. A represents an $m \times n$ matrix of coefficients assigned to each of the n variables across each m inequality equations (referred to as constraints). b represents a vector of length m of real numbers ($b \in R^m$) specifying the right hand side of the linear equations defining the constraints. Finally l and u represent vectors of integer numbers ($l, u \in Z^n$) of length n and which define the upper and lower bounds of each decision variable x. The integer variables to be estimated correspond to the discrete values that we can assign to network components (protein activities, interactions, etc.). For example for the Boolean

modelling of a signalling network, each component is allowed to either take value 0 ($l = 0$) or 1 ($u = 1$). On the other hand, the constraints can be formalised in such a way which allows the reconstruction of signalling network solutions in consistency to a set of rules we have in mind. These constraints are expressed through linear equality/inequality equations of the discrete decision variables and the solution must satisfy these constraints. Lastly, the objective function can be defined in such a way to for example minimise the discrepancies between our inferred model predictions and the data we use to train it.

**Sets & Variable**	**Description**
E	The set of protein-protein interactions e in the prior knowledge
$E^{'}$	The set of domain-domain interactions e' in the prior knowledge
Φ	The set of $NULL$ domain-domain interactions ($\Phi \subset E^{'}$) (see below for more details)
$\Theta_{e} = \Theta_{(u,v)}$	The set of domain-domain interactions e' between proteins u and v ($E^{'} = \bigcup_{(u,v)}^{E} \Theta_{(u,v)}$)
$\delta\ (v)^{+}$	The set of edges in E emergent from a node $v \in V$
$\delta\ (v)^{-}$	The set of edges in E incident to a node $v \in V$
I	The set of perturbation targets
P	The set of inferrable nodes/signalling proteins
T	The set of TF’s

$x_u \in \{0, 1\}$	Variable indicating the presence of a node u (or domain u') in the solution
$z_{e'} = z_{(u',v')} \in \{0, 1\}$	Variable indicating the presence of a domain-domain interaction e' connecting domains u' and v'
$y_e = y_{(u,v)} \in \{0, 1\}$	Variable indicating the presence of a protein-protein interaction e connecting proteins u and v in the optimal final solution

Table 1.: Set of variables used in the ILP formulation

In our case, we define as $G = (V, E)$ and $G' = (V', E')$ the set of protein-protein and domain-domain interactions from DIGGER. The goal here is to identify a subset of functional PPI's and DDI's from G and G' based on evidence from gene expression as well as differential splicing analysis. The assumption here is that two proteins are predicted to be interacting if there exist two domains, one from each protein, which are interacting.

We start first by defining the set of variables and variable sets as described in Table 1. Here all the variables can take either values 0 or 1. Depending on how the users want to handle the significance scores of the exon/domain splicing effects, signalling networks can be contextualised with LINDA following two different modes with slight differences in the ILP formulation of LINDA: the hard-constrained and the soft-constrained analysis modes:

1- Hard-Constrained Approach

The optimization goal for this case is to minimise the objective function (OF) shown in (1):

$$min\{\sum_{u \in T} \lambda_1 \cdot s_u + \sum_{(u', v') \in E'} \lambda_2 \cdot z_{(u',v')}\} \qquad (1)$$

It can be noted that the OF consists of two terms:

1. In the first term ($\sum_{u \in T} \lambda_1 \cdot s_u$) it is defined as the primary optimization goal: The network solution must include as many of the top-regulated TF's while penalising the

inclusion of the TF's which do not appear to be regulated. For that for each TF node u in G ($u \in T$) a score has to be assigned as according to the following scheme:

$$s_u: \begin{cases} = -1 & \forall u \in topTF \\ = 1 & otherwise \end{cases}$$

We define $topTF$ as the set of most regulated TF's (by default: top 50 TF's based on their absolute enzymatic activity values as defined by the normalised enrichment score values). In this case, if a TF belongs to the $topTF$ it will be assigned a score of -1, otherwise the node will assume a score of $+1$. Since the objective function is defined as a minimization function, then ILP will try to include as many of the most regulated TF's in the solution (those with a score of -1), while penalising the inclusion of the other TF's.

2. We define the second optimization goal by the second term ($\sum_{(u',v')\in E'} \lambda_2 \cdot z_{(u',v')}$) in the OF: penalising the inclusion of DDI's in the solution. Through this term, we ensure that LINDA gives us the network model with the best quality and with a minimal number of components (domain interactions). The assumption for this is that biological systems typically operate with minimal energy and with the least number of reactions to reach a specific goal.

The balance between prioritising good fit (primary objective) or sparse model (third objective) is regulated by the tunable parameters λ_1 and λ_2. By default it is set that $\lambda_1 = 100$ and $\lambda_2 = 1$. This is done this way in order to give a much higher priority to the inclusion of the top-regulated TF's in the solution network compared to the size of the network. The λ_2 is set typically to be set to a very small value in order to not affect the inclusion of any top-regulated TF in the network and to penalise the inclusion of redundant DDI's (and hence PPI's since they are interchangeable) which do not have much influence on the primary objective.

Next, the set of linear constraints which define the rules of network reconstruction are as stated in the equations $(2 - 8)$:

$$x_u = 1 \qquad u \in I \quad (2)$$

$$x_u + x_v \geq 2 \cdot y_{(u,v)} \quad e = (u,v) \in E \qquad (3)$$

$$\sum_{(u,v)\in\delta^+(u)} y_{(u,v)} \geq x_u \quad u \in P \ (4)$$

$$\sum_{(u,v)\in\delta^-(v)} y_{(u,v)} \geq x_v \quad v \in P \ (5)$$

$$y_{(u,v)} \leq \sum_{\Theta_{(u,v)}} z_{(u',v')} \qquad e = (u,v) \in E \qquad (6)$$

$$y_{(u,v)} \geq z_{(u',v')} \ \forall e' = (u',v') \in \Theta_{(u,v)} \qquad e = (u,v) \in E \qquad (7)$$

$$z_{(u',v')} = 0 \qquad e' = (u',v') \in \Phi \qquad (8)$$

The main variables in the list of constraints are x, y and z as defined in Table 1. All of these variables are defined as Boolean, meaning that they can take either a value of 0 or 1. When any of these variables take a value of 0, it means that the corresponding protein/PPI/DDI is not functional and hence will not be present in the final given solution. Otherwise, when any of these variables takes a value of 1 after optimization, that means that the protein/PPI/DDI is functional and hence it will be reported in the final solution.

The role of each constraint is explained by considering the equations $(2 - 8)$ one-by-one:

1. Through constraint 2, we define as a functional node any of the molecules in the network which is perturbed. These perturbations can be drug perturbations, ligand treatment of target receptors, etc.. In the context when we have no perturbation in our experiments, an auxiliary *Perturbation* node is automatically defined as the perturbation target. This *Perturbation* node is included in the network by connecting it with the uppermost proteins in the DIGGER database. The uppermost proteins are defined as those ones which appear to have no incoming interaction, but only outgoing ones.

2. Through constraint 3, it is ensured that a protein interaction between two nodes can potentially happen only if these two proteins (u and v) between the interaction $e = (u,v)$ are present in the final solution.

3. Through constraints 4 and 5, we ensure that every intermediary node $u \in P$ present in the solution to have at least one outgoing and one incoming protein interaction. Intermediary nodes refer to the proteins which belong to neither the perturbation target (I) and nor the TF set (T), $P = V - \{I, T\}$.

4. Through constraints 6 and 7, it is ensured that two proteins will be predicted to be interacting if there exist at least one pair of interacting domains in between the two proteins. Here, the $\Theta_{(u,v)}$ refers to the set of all interacting domains between each protein interaction pair u and v.

5. Through constraint (8), we ensure that for any DDI in the PKN whose exon/domain skipping event significance score falls within a threshold value $pValThresh$, then the corresponding domain interaction will automatically be assigned a value of 0, meaning that it is not functional and hence not present in the final solution ($z_{(u',v')} = 0$ if at least one of the interacting domains u' or v' is skipped). The set of domain interactions in which at least one of the interacting domains falls below this significance threshold set by the user is called the $NULL$ set and it has been designated as Φ (where $\Phi \subset E'$). By default $pValThresh = 0.05$.

Interaction loops present in $G = (V, E)$ can be the cause of inferring self-regulating cycles in the network solutions and which are independent from the upstream source (i.e. 'Perturbation' node). In order to prevent self-activating cycles between proteins to appear in the network solutions, additional loop constraints have been adapted from (Melas et. al. 2015).

We start by introducing the variable $d_u \geq 0$ assigned to each protein node u in DIGGER. The implemented loop constraints are then as follows:

$$x_u \leq d_u \quad (9)$$

$$d_v \geq d_u + 1 - M + y_{(u,v)}M \quad (10)$$

$$d_u \leq M \quad (11)$$

Where M ($M = 1001$ by default) is a sufficiently large number and node v is the target of the source node u.

In this way, through the constraints in $(9 - 11)$ we ensure that a feedback loop will not be feasible since for example if we assume that a loop is conversed in the solution for source node u and target node v, then we should have $d_v > d_u$. However, since in a loop we also have that node v is upstream of node u, then we will also have that $d_u > d_v$. This creates two inequality contradictions and thus the presence of loops in the network solution will become infeasible.

<u>2- Soft-Constrained Approach</u>

The ILP formulation of LINDA when using the soft-constrained mode is almost the same as the formulation described above with only a few minor differences.

When wanting to contextualise protein interaction networks in the soft-constrained approach, users must set $pValThresh = NULL$. In this case, the λ_2 parameter of the objective function (equation (1)), instead of taking a discrete value it will be assigned a continuous value based on the significance score associated to the skipping event of each domain present in the background network:

$$min\{\sum_{u \in T} \lambda_1 \cdot s_u + \sum_{v' \in V'} \lambda_2 \cdot x_{v'}\} \qquad (1')$$

The continuous score is simply assigned by the logarithmic value of the significance score of the exon skipping event. In this case, for domains which we are more confident that have been skipped (as reflected by lower significance p-value scores) we will have higher logarithmic values and thus penalise the inclusion of such domain to the network solution compared to other less confident cases.

Since we are including the splice effect on the objective function, for the soft-constrained application we can then remove the hard constraints defined in (8).

S-Text-2: LINDA Solvers. LINDA supports the following Linear Programming solvers in order to perform the network reconstruction procedure:

- **CPLEX:** The IBM ILOG CPLEX Optimization Studio (https://www.ibm.com/products/ilog-cplex-optimization-studio) is a commercial solver which is freely available through Academic Initiative. We strongly encourage using cplex to solve the LINDA problems since it offers several advantages compared to other solvers as well as all the examples provided have been analysed with CPLEX. The main advantages of CPLEX consists of its ability to efficiently solve large-scale problems (such as we typically have with LINDA), it can enumerate multiple alternative solutions as well as it offers a wide range of parameters for controlling the optimisation procedure.
- **COIN-Cbc:** The COIN-Cbc (https://github.com/coin-or/Cbc) solver is a free and open-source mixed integer programming solver written in C++. The solver is able to solve large-scale linear problems efficiently however in LINDA it does not enumerate multiple alternative solutions.
- **lpSolve:** The lpSolve (https://cran.r-project.org/web/packages/lpSolve/index.html) is a freely available (under LGPL 2) software/R-package for solving linear, integer and mixed integer programs. The solver is only able to solve small-case toy examples and it is not capable of enumerating multiple alternative solutions.

S-Tab-1: Parameter settings. *runLINDA()* is the main function which the LINDA package uses to analyse and reconstruct the networks. There are a number of parameter settings implemented in this function and through which we can control the quality and diversity of solutions that are retrieved. A list of such parameters has been provided in Table 2.

Parameter	Description
lambda1	The penalization term of the primary objective in the objective function - TF inclusion. This penalty factor is suggested to be set to a higher value compared to other penalty parameters in order to strongly penalise the inclusion of not significantly regulated TF's. By default, lambda1=100.
lambda2	The penalization term of the secondary objective of the objective function - DDI inclusion (size penalty). This penalty factor is suggested to be set to a lower level compared to lambda1 parameters in order to remove redundant domain-domain interactions which can explain the regulation of downstream TF's. By default, lambda2=1.
pValThresh	(optional) a p-value threshold to indicate which are the significantly skipped exons. Those exons in which the corresponding FDR value (from as.input) falls below the pValThresh parameter, are considered to be skipped and such information will be integrated in the AS-constraints of the ILP formulation. By default, pValThresh=0.05. In case users want to run the soft-constrained mode of LINDA, they can simply set this parameter to NULL (pValThresh=NULL).
mipgap	CPLEX parameter which sets an absolute tolerance on the gap between the best integer objective and the objective of the best node remaining. When this difference falls below the value of this parameter, the mixed integer optimization is stopped. By default, mipgap=0.

relgap	CPLEX parameter which sets a relative tolerance on the objective value for the solutions in the solution pool. Solutions that are worse (either greater in the case of a minimization, or less in the case of a maximization) than the incumbent solution by this measure are not kept in the solution pool. By default, relgap=0 (or 0 percent), meaning that only best solutions can be included as incumbent solutions (if that solution has been reached).
populate	CPLEX parameter which sets the maximum number of mixed integer programming (MIP) solutions generated for the solution pool during each call to the populate procedure. Populate stops when it has generated the amount of solutions set in this parameter. By default, populate=500.
nSolutions	The number of solutions to be provided by LINDA. By default, nSolutions=100.
timelimit	CPLEX parameter which sets the maximum optimization time in seconds. By default, timelimit=3600 (3600 seconds or 1 hour).
intensity	CPLEX parameter which controls the trade-off between the number of solutions generated for the solution pool and the amount of time or memory consumed. Values from 1 to 4 invoke increasing effort to find larger numbers of solutions. Higher values are more expensive in terms of time and memory but are likely to yield more solutions. By default, intensity=1.
replace	CPLEX parameter which designates the strategy for replacing a solution in the solution pool when the solution pool has reached its capacity. The value 0 replaces solutions according to a first-in, first-out policy. The value 1 keeps the solutions with the best

	objective values. The value 2 replaces solutions in order to build a set of diverse solutions. By default, replace=1.
threads	Number of threads to be used for optimisation. By default, threads=0 (let the solver choose).

S-Tab-2: A table of perturbation targets in K562 clustered to each of their specific phenotypes and their corresponding GO pathway sets.

Perturbation Target	Associated Phenotype	Corresponding GO
HNRNPA2B1	Histone Acetylation	GO:0016573
HNRNPC	NuA4 histone acetyltransferase complex	GO:0035267
MAGOH	Non-sense Mediated Decay	GO:0000184
HNRNPU	Spliceosome	GO:0000398
NCBP2	mRNA Capping	GO:0006370
PABPN1	Exosome and mRNA turnover	GO:0000178
PAPOLA	Mediator Complex	GO:0016592
PCBP1	COP9 Signalosome	GO:0008180
POLR2G	Nucleotide Excision Repair	GO:0006289
PPIL4	Spliceosome	GO:0000398
PRPF6	Spliceosome	GO:0000398
PRPF8	Spliceosome	GO:0000398
PUF60	Spliceosome	GO:0000398
SF1	NuA4 histone acetyltransferase complex	GO:0035267
SRSF1	Mediator Complex	GO:0016592
SMNDC1	Spliceosome	GO:0000398

S-Tab-3: Antibodies

Target	Vendor	Cat No	Dilution
Erk1 (MAPK3)	Abcam	ab32537	1:5,000
HNRNPK	Santa Cruz	sc-28380	1:500
SREBF2	Thermo Fisher Scientific	PA1-338	1:500
HNF4A	Thermo Fisher Scientific	MA1-199	1:1,000
